# A Qualitative Study to Explore Perspectives Regarding the Use of Low Field Magnetic Resonance Imaging (LFMRI) Scanners, Within Dementia Diagnosis Pathways in the United Kingdom

**DOI:** 10.1192/bjo.2025.10216

**Published:** 2025-06-20

**Authors:** Rachel Rice, Ursula Shepherd, Lisa Dikomitis, Jamie Harper, Joanne Rodda

**Affiliations:** 1 KMPT, Kent, United Kingdom; 1 KMMS, Canterbury, United Kingdom

## Abstract

**Aims:** The national emphasis on improving rates and timeliness of dementia diagnosis is dependent on accessibility of investigative tools. Through locally accessible, point-of-care brain scans, LFMRI has the potential to improve the experience of dementia assessment pathways and time to diagnosis, and to reduce inequalities in access to dementia assessment.

The aim of this qualitative research was to explore perspectives regarding the use of LFMRI scanning within dementia diagnosis pathways, within communities where it may have the greatest impact. We also aimed to learn more about views regarding future LFMRI research, including priorities, concerns and potential facilitators and barriers to participation.

**Methods:** The qualitative design incorporated focus groups and interviews with individuals with dementia and their carers. The study took place within urban, rural and coastal communities in Kent. 35 participants took part in either a focus group (n=20) or interview (n=15) with an average age of 72 years. Focus groups and interviews were recorded and transcribed verbatim for thematic analysis using NVivo software.

**Results:** Participants described both positive views as well as caution about the use of this new investigative tool. Five subthemes were identified: access to local neuroimaging, improvement of assessment pathways, accuracy of LFMRI, concerns about expense to the NHS and engagement in future LFMRI research.

Participants were optimistic about the potential of LFMRI within dementia diagnosis pathways. They valued the possibility of access to local scanners and the benefit this would have on timely diagnosis with improved diagnostic pathways. However, there were concerns about the accuracy of these scanners and the cost implications within the NHS.

**Conclusion:** While the technology will not mean the replacement of traditional MRI scans, LFMRI may have the potential to improve dementia diagnosis pathways. LFMRI may be a lower cost alternative to help to reduce wait times while improving access to pathways for those in underserved communities. This would be welcomed by people with dementia and their carers, if there was trust in the accuracy of the scans. Participants expressed willingness to be involved in further research using LFMRI scanners to support improvements in the dementia diagnostic pathways for others.

